# Combustion-Generated Nanoparticulates in the El Paso, TX, USA / Juarez, Mexico Metroplex: Their Comparative Characterization and Potential for Adverse Health Effects

**DOI:** 10.3390/ijerph2006030007

**Published:** 2006-03-31

**Authors:** L. E. Murr, K. F. Soto, K. M. Garza, P. A. Guerrero, F. Martinez, E. V. Esquivel, D. A. Ramirez, Y. Shi, J. J. Bang, J. Venzor

**Affiliations:** 1 Department of Metallurgical and Materials Engineering, The University of Texas at El Paso, El Paso, TX 79968, USA; 2 Department of Biological Sciences, The University of Texas at El Paso, El Paso, TX 79968, USA; 3 Department of Mechanical and Industrial Engineering, The University of Texas at El Paso, El Paso, TX 79968, USA; 4 Southwest Allergy and Asthma Associates, P.A., 10501 Vista del Sol, Suite 114, El Paso, TX 79925, USA

**Keywords:** TEM analysis, Carbonaceous nanoparticulates, Carbon nanotube aggregates, Clinical and city-wide surveys, Asthma incidence, Cytotoxicity assays

## Abstract

In this paper we report on the collection of fine (PM_1_) and ultrafine (PM_0.1_), or nanoparticulate, carbonaceous materials using thermophoretic precipitation onto silicon monoxide/formvar-coated 3 mm grids which were examined in the transmission electron microscope (TEM). We characterize and compare diesel particulate matter (DPM), tire particulate matter (TPM), wood burning particulate matter, and other soot (or black carbons (BC)) along with carbon nanotube and related fullerene nanoparticle aggregates in the outdoor air, as well as carbon nanotube aggregates in the indoor air; and with reference to specific gas combustion sources. These TEM investigations include detailed microstructural and microdiffraction observations and comparisons as they relate to the aggregate morphologies as well as their component (primary) nanoparticles. We have also conducted both clinical surveys regarding asthma incidence and the use of gas cooking stoves as well as random surveys by zip code throughout the city of El Paso. In addition, we report on short term (2 day) and longer term (2 week) in vitro assays for black carbon and a commercial multiwall carbon nanotube aggregate sample using a murine macrophage cell line, which demonstrate significant cytotoxicity; comparable to a chrysotile asbestos nanoparticulate reference. The multi-wall carbon nanotube aggregate material is identical to those collected in the indoor and outdoor air, and may serve as a surrogate. Taken together with the plethora of toxic responses reported for DPM, these findings prompt concerns for airborne carbonaceous nanoparticulates in general. The implications of these preliminary findings and their potential health effects, as well as directions for related studies addressing these complex issues, will also be examined.

## Introduction

In spite of efforts worldwide to reduce atmospheric particulate levels, particularly airborne anthropogenic particulate matter (PM), the prevalence of allergy-related respiratory diseases is increasing dramatically, especially in industrialized countries [1]. Epidemiology has demonstrated that susceptible individuals are being harmed by atmospheric PM levels comparable to current U.S. air quality standards which apply to PM less than 10 μm in diameter (PM_10_). PM smaller than 0.1 μm (≤100 nm) actually dominates the total number of particulates especially in urban aerosols, and such ultrafine PM (or nanopaticulates) are increasingly demonstrated to be toxic, [2–4], and to pose considerable health risks; including asthma complications, chronic bronchitis, respiratory tract infections, ischaemic heart diseases, and mortality [5, 6]. In the face of mounting evidence that nanoparticulates are associated with adverse respiratory health in particular, the appropriateness of filter samples or mass concentration (gravimetric) studies of atmospheric PM as a measure of potential health effects becomes questionable [7, 8]. The necessity to consider other PM characteristics such as actual PM number concentration, particle sizes and size distribution, morphology, detailed chemical speciation, and other characteristics of individual airborne particles correspondingly is becoming a paramount issue.

Filter samples in particular provide only time-averaged PM properties while individual PM compositions contain information that is important for both toxicological studies and source apportionment. Buseck and Posfai [9] have also recently noted that it is the individual chemical species of atmospheric PM that affect the radiative balance and climate as well as visibility and health. Paraphrasing the work of Michaels [10], they asserted that “interpreting environmental and health effects of aerosols from bulk rather than individual-particle analyses is like interpreting mortality reports in a war zone from bulk air borne lead concentrations rather than from bullets”. Buseck and Posfai [9] concluded from studies of individual aerosol particles that they are ubiquitous in the troposphere and exert an important influence on global climate. Minerals or mineral PM comprise the dominant mass fraction of the atmospheric aerosol burden. In this context, Andreae [11] has estimated that mineral aerosols compose roughly 16.8 Tg of the mass abundance of the global atmospheric aerosol while industrial sources, including soot, compose 1.4 Tg of the mass abundance. However a more recent assessment of aerosols containing soot or black carbon (BC) estimates the global mass abundance of BC to be 7 Tg; with annual U.S. emissions of 0.32 Tg of BC in contrast to 1.2 Tg of BC for China [12]. This does not necessarily or specifically include other carbonaceous PM such as wood combustion PM or diesel particulate matter (DPM), which is similar in size and morphology, and primarily compose the fine PM (PM_1_) and nano-PM (PM_0.1_) regimes [13].

Warheit [14] has recently pointed out that the total lung toxicity database for comparing the effects of fine (∼0.1 to 2.5 μm) versus ultrafine (<0.1 μm) PM consists primarily of studies on three particle types: titanium dioxide (TiO_2_), black carbon (BC), and diesel particles or diesel particulate matter (DPM). Some comparisons have also included fine and ultrafine silica (SiO_2_) PM [15]. Moreover, diesel particles (or DPM) usually include adsorbed or otherwise complexed polycyclic aromatic hydrocarbons (PAHs) which have not been separated from the actual DPM. However, Nikula, et al. [16] concluded that the organic fraction of DPM may not play an important role in its carcinogenicity in rats, while Heinrich, et al. [17], and Brightwell, et al. [18] demonstrated that a pulmonary carcinogenic response in rats requires the presence of the DPM, not diesel exhaust gases. In a related study, Heinrich, et al. [19] demonstrated that the carbon core of DPM was mainly responsible for the occurrence of diesel engine exhaust-related lung tumors in rats, and the role of the attached PAHs was probably of minor importance in the rat lung. Recent work by Gerde, et al. [20] has concluded that in the case of inhalation exposure to DPM with adsorbed PAHs, critical exposures of lung tissues are likely dominated by the desorbed PAH fraction deposited on the lining layer of the conducting airway. Consequently, it is unknown whether the range of toxicity and related health effects [3, 21] are a consequence of the PAHs or the particulate sizes or morphologies, which are complex, branched aggregates of individual carbon or carbonaceous spherules ranging in size from 10 to 50 nm in diameter [13]. Katrinak, et al. [22] have earlier characterized DPM in an urban aerosol as carbonaceous fractal aggregates containing as many as 1800 individual nanospherules. Katrinak, et al. [23] noted in subsequent work that the Phoenix (urban) coarse aerosol was dominated by soil-derived materials (minerals) and carbonaceous PM; with over 60% of the mass abundance of fine PM (≤2 μm particle diameter) comprised of carbon or carbonaceous matter ; half (∼30%) characterized by elemental or graphitic carbon. This compares with roughly 20% of carbon or carbonaceous matter composing coarse (PM_10_) aerosols [24]. Correspondingly, the fine and ultrafine PM regimes do not contain significant amounts of sulfates, nitrates or other secondary particulates, especially in the nanoparticle regime; including DPM [25].

Recent work by Murr, et al. [26–28] has demonstrated that a significant fraction of the airborne nano-PM is crystalline, and this includes crystalline or quasi-crystalline forms of carbon such as carbon nanotubes and other fullerene polyhedra. In fact, carbon nanotube and other fullerene polyhedra are created in aggregates or aggregated PM in a variety of common combustion exhausts, including natural gas and propane burning, both indoor and exhausting to the outdoor environment. However, it is unknown what fractions of these aggregated nanoparticles are present in the total airborne carbon or carbonaceous PM concentration or mass abundance. It is known that in the fine and ultrafine PM regimes crystalline PM is a greater health hazard than amorphous PM [15]. Furthermore, a propensity of fine and ultrafine PM appears to be aggregated and these aggregates may disaggregate, fragment, or redisperse when inhaled, allowing for exaggerated diffusion of nanoparticle components into deep lung tissue, or transmigration to the interstitial anatomical compartment of the respiratory system [29, 30].

Recent studies by Lam, et al. [31] using intratracheal instillation of carbon nanotube material (single-wall carbon nanotube ropes prepared by metal catalysis) concluded that if single-wall carbon nanotubes reach the lung, they can be more toxic than quartz. Shvedora, et al. [32] found that dermal exposure of humans to unrefined single-wall carbon nanotubes can result in accelerated oxidative stress and toxicity in the skin of exposed workers. Warheit, et al. [33] more recently observed transient inflammatory and cell injury effects in rat lungs instilled with single-wall carbon nanotube rope particulates while Murr, et al. [34], and Soto, et al. [35] have observed a cytotoxic response for murine macrophage cell-line assays with single-wall carbon nanotube ropes, several multi-wall carbon nanotube aggregate materials, and nanoparticulate black carbon; which was essentially the same as that observed for chrysotile asbestos nanotube particulates. In this latter work [35], one of the multi-wall carbon nanotube aggregate materials was demonstrated to be microstructurally identical to the multi-wall carbon nanotube aggregates observed in the ambient air [28, 36], and therefore a probable surrogate.

## The Nanoparticulate Paradigm

Oberdörster, et al. [37] and Seaton, et al. [30] were among the first to suggest an ultrafine particle hypothesis wherein such particles would demonstrate enhanced inflammatory responses as well as more chronic effects in lung systems of animals and humans. While the fundamental reasons or mechanisms for this are unclear, there is now a plethora of data over at least the last decade or more that compellingly demonstrates that ultrafine or nanoparticulates induce greater, adverse pulmonary responses than fine or coarse particulates [4, 14, 15, 37, 38–40]. This is especially true for several specific nanoparticulate model types: TiO_2_, black carbon, and silica [14, 16, 38]. However it is only black carbon (BC) and silica which are prominently ubiquitous in the global atmosphere [12].

The health of individuals, especially the susceptible (older and chronically ill individuals) is being compromised by ambient PM at levels below or comparable to current air quality standards based on mass loadings. Particle mass, which has been the focus of the contemporary paradigm for PM analysis and health effects, is likely to be a surrogate for the real agents which must lie somewhere in the nanoparticulate regime. There is an inherent uncertainty introduced by the interconversion of particle mass concentration data. For example the nanoparticles in the ultrafine transient mode of diesel engines represent only 0.1 to 1.5% of particle mass, but roughly 37 to 97% of the particle number; with typical DPM mass concentrations of 15 to 30 mg/m^3^ [41]. This is in contrast to normal (ambient outdoor), measured, mass concentrations of nanoparticulates (or ultrafine PM) ranging from ∼0.8 to 1.6 μg/m^3^ [42] and corresponding nanoparticle number concentrations of 1 to 5×10^10^/m^3^.

Although the contribution of ultrafine or nanoparticulates to particle mass is very small, they are present in the air in very large numbers. Fixed site, total mass particulate monitors disproportionately represent the largest-size particles and do not effectively measure the most important particulate size fraction associated with adverse health effects [43]. Furthermore, bulk analyses of particle mass collections do not identify individual species which may have a unique connection to specific health outcome issues or contribute to the underlying biological mechanisms of particulate toxicity. Consequently, a paradigm shift needs to occur where analytical strategies focus on the collection and examination of specific particles or particle aggregates in the nanoparticulate regime: their chemistry, size, morphology and crystallinity in particular. The development of simple cytotoxicity assays to assess potential respiratory or other health links would also be a useful diagnostic for ambient nanoparticles as well as those produced for nanotechnology applications. Long term risk assessment for ubiquitous anthropogenic nanoparticulate species and source identification will also drive new epidemiological approaches as well as etiological considerations.

In the present study, we examined several hundred individual, airborne (outdoor) particulates collected by thermal precipitation onto coated transmission electron microscope (TEM) grids which were observed in an analytical TEM. The main objective of this particulate analysis was to deter mine the frequency, size distribution, and morphology of aggregated PM, and to characterize the nanoparticle components and their crystallinity, and chemical speciation; or elemental occurrence. More importantly we were looking for common or ubiquitous PM species, especially anthropogenic, carbonaceous species, which might point to new directions in etiologic, epidemiologic, or more specific toxicologic studies; especially for nanomaterials or nano-PM in the environment. In addition, we characterized and compared DPM, burning tire particulate matter (TPM), and wood (combustion) particulate matter (WPM)). These carbonaceous nanoparticulate species were also compared with carbon nanotube aggregates. Carbon nanotube aggregates were also collected and analyzed in the indoor air, particularly kitchens. Short-term (days) and long term (weeks) cytotoxicity assays were also conducted for surrogate carbon nanotube aggregates and black carbon (BC), and both clinical and general population surveys were conducted in El Paso to begin to assess the propensity of gas stove use and exposure as well as the possible connection with asthma incidence and associated respiratory issues.

## Experimental Methods and Materials

### Outdoor PM Collection and Analysis

Airborne PM examined in this study was collected over a nearly 2 year period from a wide range of outdoor areas in El Paso, Texas, USA. These included street corners, parking lots, truck stops, freeway locations, and other more specific location s corresponding to characteristic source emissions such as bus garages, roof top natural gas combustion exhausts, and areas proximate to specific industrial operations utilizing natural gas and exhausting into the atmosphere. Collections were also made for wood combustion and burning tires.

Collections were made using a thermal (or thermophoretic) precipitation device described in detail elsewhere, including calibration procedures [44, 45]. In this device, a temperature gradient is created between fine and ultrafine PM drawn across a hot wire, which drives efficient adsorption of the PM onto coated, 3 mm TEM grids placed on an ice-water-cooled copper block located below the hot wire. The TEM grids were either 100 mesh nickel grids coated with ∼35 nm layers each of formvar and carbon, or 200 mesh copper grids coated with ∼35 nm layers each of formvar and silicon monoxide. The carbon/formvar coated grids were often carefully inspected to assure no adsorbed PM artifacts which were especially important in the collection of carbon or carbonaceous PM, and these carbonaceous collection regimes were also often replicated using the silicon monoxide/formvar-coated grids. The PM collections from or associated with carbon nanotubes or related carbon nanoparticle aggregates employed the silicon monoxide/formvar coated grids exclusively to avoid artifacts. Collections were usually conducted for 0.5 h periods 1.5 m above ground level, except in sampling exhausts or other specific source regimes where collection times were reduced to 15 minutes. Sample collections were normally made in calm conditions (no wind speed) except for special collections or sites to be noted.

For normal operation of the thermal precipitator the air flow velocity into the device was measured to be 30 m/min. Utilizing a calibrated collection efficiency of ∼0.9 (∼90%) [45], the particulate abundance or number concentration (N)/m^3^ could be approximated by measuring the number of particulates collected on a measured area of TEM grid (A in m^2^): N/m^3^ ≅ N/0.9A (30 m/min)(collection time in minutes). Collecting cigarette smoke for 30 min. produced a particulate (number) concentration of ∼10^10^/m^3^; with a wide distribution of spherical, car bonaceous particle diameters.

The experimental grids with collected PM were examined in a Hitachi H-8000 analytical TEM fitted with a Noran energy-dispersive (X-ray) spectrometer (EDS), and a goniometer-tilt stage. The accelerating potential was 200 kV for the TEM operation which involved bright and dark-field imaging of collected particulates utilizing their corresponding selected-area electron diffraction (SAED) pattern which also provided information about the crystallinity, degree of crystallinity, or other specific crystallographic features [46]. The EDS analysis of individual PM provided qualitative information about the chemical speciation in terms of the relative intensities of elemental components, with an accuracy of ∼ 1 weight percent.

### Indoor (Kitchen) Carbon Nanotube Aggregate Collection and Analysis

Utilizing the same procedures as outlined above for outdoor particulate collection and sampling, kitchens with gas stoves (propane and natural gas (∼96% methane)) were sampled with the thermal precipitator located ∼0.5 m above the gas flame, or at various other kitchen locations. These samples were collected on silicon monoxide/formvar-coated 200 mesh Ni TEM grids which were observed in the TEM as outlined above. Approximately 5 kitchen locations using propane and 5 locations using natural gas were sampled.

### Cytotoxicity Assays of Surrogate Nanomaterials

The surrogate materials (BC-vulcan XC-72 and Rosseter MWCNT aggregates) and chrysotile asbestos as a test reference material as described in detail elsewhere [34, 35], were suspended in a stock solution at 5 μg/mL in dimethyl sulfoxide (DMSO). DMSO is a very general solvent which can assure suspension of even notable hydrophobic substances. The test materials in the DMSO were vortexed (stirred) in a Vortex Genie 2 for 1 minute to assure a uniform suspension. Murine alveolar macrophages (RAW 267.9 cells: courtesy of Kenneth S. K. Tung at the University of Virginia Health Sciences Center) were cultured in 96-well flat-bottom plates (50,000 cells/well) in the presence of decreasing concentrations of compound (starting at 10 μg/mL with 11 doubling dilutions thereafter). The confluency level of the cells prior to treatment was 80%. The biological assessment is based on viability and not cell growth. Controls were incubated with equivalent dilutions of vehicle (DMSO) and with neither vehicle nor compound. The cells were cultured in DMEM, 10% FCS, 5 × 10^−5^ M 2-Me, penicillin, streptomycin, and 2 mM glutamine at 37°C in 5% CO_2_. After 48 hrs. of incubation, 20 μL of MTT (3-(4,-dimethylthiazol-2-yl)-2,5-diphenyltetrazolium) (5 μg/mL in H_2_O) (Sigma-Aldrich Co., St. Louis, MO) was added and the cells were incubated for an additional 4–6 hrs. at which time 180 μL of supernatant was removed and 50 μL of lysis buffer, containing 10 N HCl in isopropanol, was added. After several minutes, the MTT crystals formed were solubilized with gentle pipetting and the content of dissolved MTT crystals was measured with a Molecular Devices VersaMax tunable microplate reader set at 50 nm (van de Loosdrecht, et al., 1994). Cell viability assessments or mitochondria/activity of living cells were made by measuring the relative absorbance or optical density (O.D.) for mitochondrial dehydrogenase-transformed formazan (or color product). The experiments were replicated three times and the data were graphically presented as means ± standard errors of the means. All of the 96-well systems were observed to be optically transparent throughout, assuring no false readings due to flocculation of the suspended nanomaterials.

For long-term exposure assays (168 and 336 h) the test materials were suspended in a stock solution of 2.5 μg/mL DMSO and vortexed as described above. The murine macrophages were cultured in a 12-well flat bottom plate in duplicate (10^5^ cells/well for 1 week (168 h) exposure and 3 × 10^6^ cells/well for the 2 week (336 h) exposure; with 1 μg/mL concentration for the test materials. The cells were cultured in DMEM and new media and test material were added every other day. After 1 or 2 weeks the cells were collected and counted by trypan blue exclusion.

### Clinical Asthma Patient Data and El Paso City-Wide Data Collection

Asthma patient data was collected by having clinical patients fill out a 2-page questionnaire randomly selected at the time of routine clinic/office visits during 2003–2005. The randomness of those completing the questionnaire also contributed to the overall sample randomness. Principal questions included whether or not patients were currently and continuously exposed to kitchen gas stoves. Asthma categories were designated severe, moderate, and mild as described in earlier surveys [34], but the analysis included all as a group of asthmatics. There were 38 respondents in the data set which was biased to include only asthmatics.

The El Paso City-wide survey instrument included similar questions to the clinical survey but was administered by personal interview on the University of Texas at El Paso campus. Respondents were asked whether they were or considered themselves to be asthmatic, but no specific category of asthma was identified. The data set contained 200 respondents distributed throughout the city, almost evenly split between male and females. Ages in the City-wide survey ranged from 3 to 83 years of age in contrast to the smaller clinical survey where ages ranged from 7 to 89 years of age.

## Results

### Ambient (outdoor) Nanoparticulate Analysis

**Figure 1:**
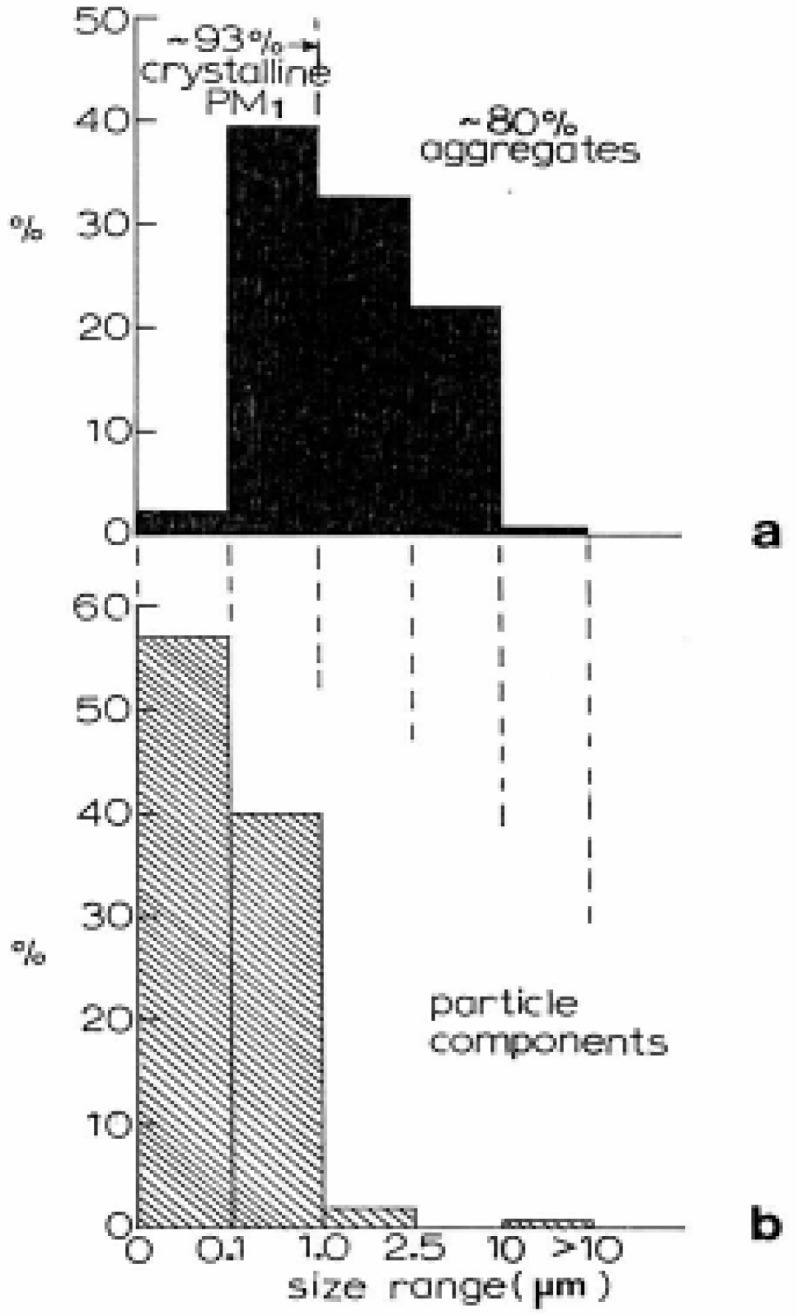
(a) PM (average) size (dp) range versus PM abundance as a percent of the total PM analyzed. Note that 93% of PM, were crystalline while 80% of all PM were aggregates. (b) Primary particle component sizes (size ranges) versus percent of the aggregated PM in (a). Note that >95% of all primary particles were PM_1_; 57% were PM_0.1_. PM collected in the El Paso, TX, USA outdoor air.

Figure 1 summarizes the particulate sizes and size distributions for PM collected throughout the city of El Paso, TX (USA) over a 2-year period (outdoors). This PM regime represents only completely characterized PM (which includes TEM imaging (bright and dark-field), SAED pattern analysis, and EDS analysis). The PM size is the mean (or average) geometrical particulate diameter (d_p_) ((the maximum (projected) diameter + minimum diameter)/2). This is effectively the equivalent particle diameter or physical diameter ; and often a close approximation of the aerodynamic diameter (which can be 10 to 30% smaller) [47]. The data in Fig. 1 represent 225 fully analyzed outdoor particulates where, as shown in Fig. 1(a), essentially 93% of PM_1_ were crystalline, while 80% of all PM were aggregates. More than 40% of the PM summarized in Fig. 1(a) was PM_1_ (d_p_ ≤ 1 μm); 98% was PM_10_. The average PM diameter in Fig. 1(a) (effectively the projected, aggregate diameters) was ∼1.5 μm. Figure 1(b) illustrates that essentially 58% of all primary particles composing these aggregates were ≤0.1 μm (≤100 nm) in diameter; or PM_0.1_. Ninety-seven percent were PM_1_.

**Figure 2:**
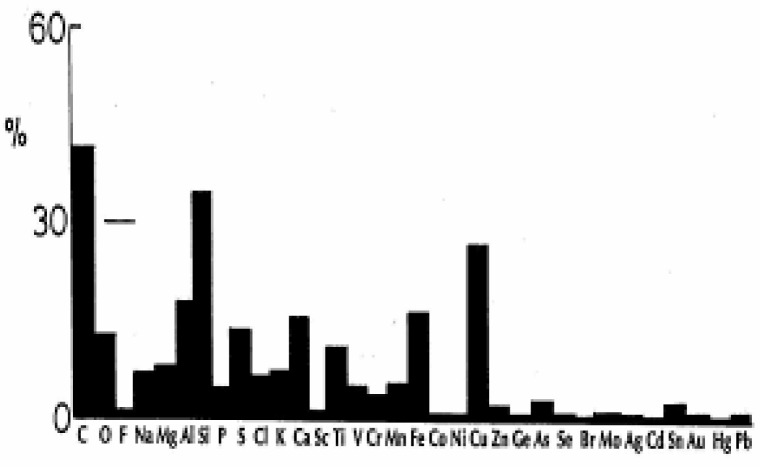
Elemental occurrence as a percent (%) of all collected PM in Fig. 1(a). Elements <1% not shown include W.

Figure 2 summarizes the frequency of occurrence (as a percent of the total) of chemical elements detected in these 225 particulates (Fig. 1(a)) from corresponding EDS spectra. It can be noted that 42% of the PM contained carbon, 33% Si, 27% Cu, 15% Al, 13% Fe, 12% Ca, and 10% S. About half (∼10%) of the carbonaceous particulates were identified as carbon nanotube aggregates while the balance were DPM or related carbonaceous aggregates, including carbonates (∼5%). It will be shown later that DPM cannot be distinguished from WPN or TPM which are often found in the El Paso ambient air.

In a study of elemental compositions for a large number of individual particles in the aerosol of Phoenix, Arizona (USA) nearly a decade ago using EDS analysis, Katrinak, et al. [23] observed that the aerosol was dominated by soil-derived materials (minerals) and carbonaceous particles. In fact, more than 60% of the PM_2.5_ regimes were carbonaceous: 32% of PM_2.5_ was organic carbon while 30% were elemental carbon. Half (50%) of the coarse fraction (PM_2.5_ to PM_10_) contained Si while only 3% of the PM_2.5_ (≤2.5 μm) contained Si [23]. In a previous study, Katrinak, et al. [22] also observed that about half of the total carbon fraction in the same location was diesel particulate matter (DPM) while the remaining carbon fraction was not specifically identified.

A more recent study of the character of individual airborne minerals and related aerosols utilizing TEM analysis as in this investigation noted that, consistent with related studies as illustrated above, a significant fraction of the aerosol particle burden consisted of minerals such as clays, quartz, calcite, gypsum, etc. A significant observation even over the Southern Ocean atmosphere was soot PM aggregated with sulfates, but this was not necessarily a ubiquitous species [97] and these aggregates were neither observed in the Phoenix, Arizona (USA) aerosol [23] nor in our studies reported herein.

We have already illustrated many of the PM represented by the data summarized in Figs. 1 and 2, especially the mineral species and DPM in previous publications [13, 26, 28, 41]. Although DPM is always observed at expected sources such as truck stop areas and other locations with heavy diesel vehicle traffic, it does not always appear in the wide range of general outdoor sampling. Silicon and iron are also expected mineral species (Fig. 2) with iron oxides, especially magnetite and hematite common in desert region sand mixtures. Calcium as calcite or more commonly aragonite (CaCO_3_) is a common PM component since it is produced in evaporative cooling which is common city-wide in El Paso; especially during the summer months.

A number of disparate samples contained small aggregates of TiO_2_ [13, 26]. While also not necessarily ubiquitous, these TiO_2_ crystalline aggregates are interesting PM especially since identically appearing nanocrystals were observed by TEM in examining the lungs of the Tyrolean ice man, a 5300 year-old mummy found preserved in alpine glacier ice [48]. Moreover, since such nanoparticles have been observed in human lungs in antiquity, it is unlikely this atmospheric PM can be linked to modern anthropogenic sources in any way that would suggest contemporary health connections

### TEM Characterization of Outdoor Carbon Nanotube Aggregates

**Figure 3:**
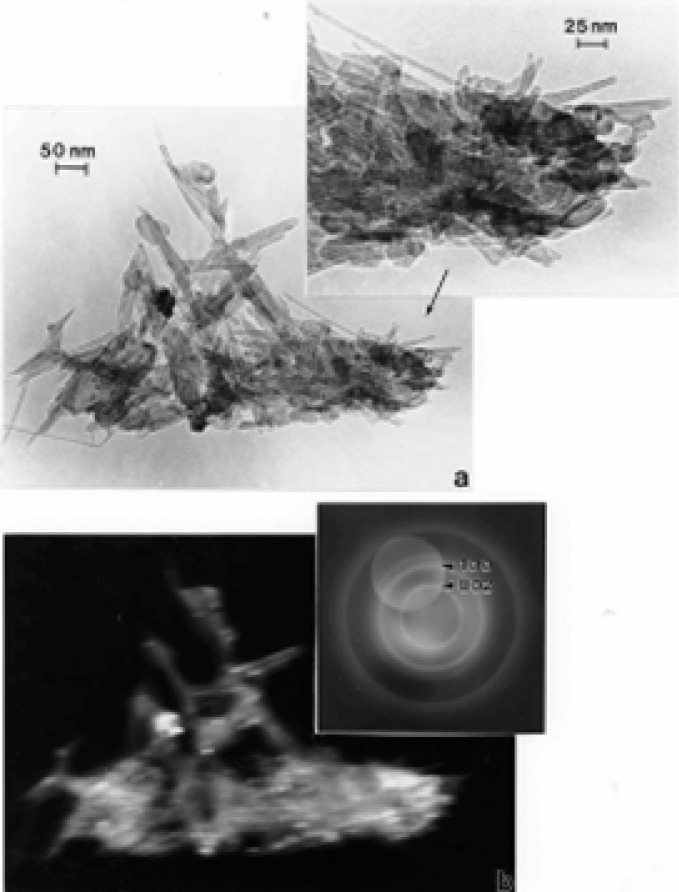
Carbon nanotube and related carbon (fullerene) nanoparticle aggregate in the El Paso, TX, USA outdoor air. (a) Bright-field TEM image. (b) Dark-field TEM image using the diffracted regime within the objective-aperture double exposure in the SAED pattern insert. Prominent hexagonal graphite (a = 0.25 nm, c = 0.67 nm) diffraction rings are indicated in the SAED pattern insert.

**Figure 4:**
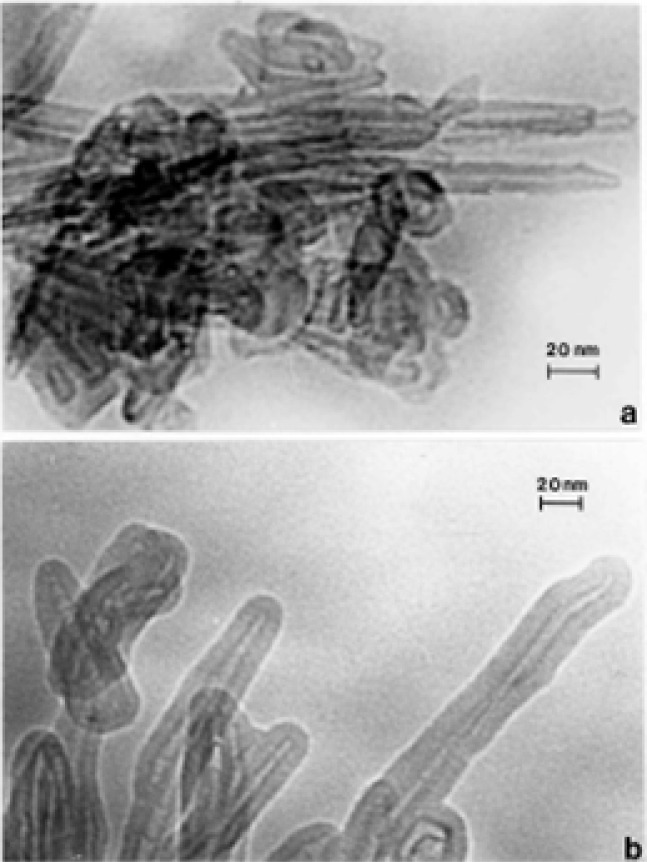
Carbon nanotubes and other carbon nanocrystal polyhedra (a) composing PM aggregates collected on the University of Texas at El Paso, TX, USA campus. Note in (b) the closed multiwall carbon nanotube structures and other fullerene morphologies readily observable at the aggregate edges. The collection site was roughly 100 m from a natural gas-burning power plant.

**Figure 5:**
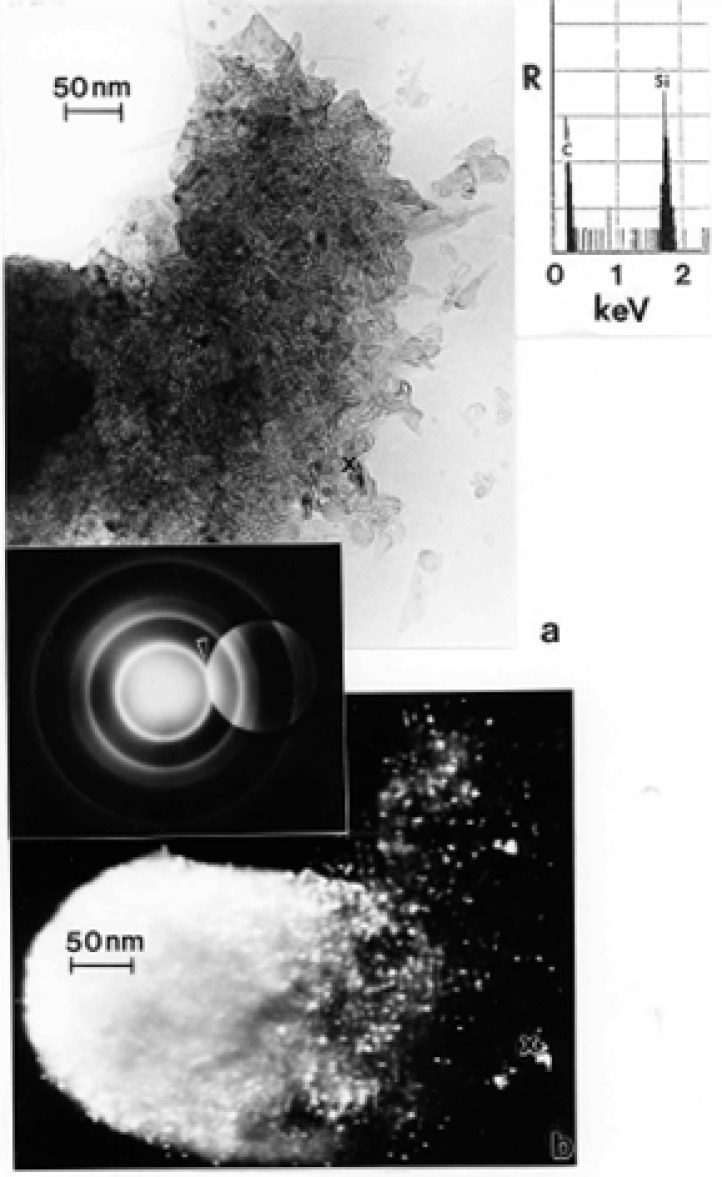
Complex aggregate composed of a mixture of carbon nanotube and related carbon nanoparticles and silica (SiO_2_) nanocrystal particles. (a) Bright-field TEM image with corresponding EDS spectrum showing C (Kα) and Si (Kα) peaks. The oxygen peak is suppressed in the X-ray emission competition. (b) Dark-field TEM image slightly shifted from (a) showing predominantly SiO_2_ strongly diffracting nanocrystals (reference X). The principal diffraction regimes marked 1 and 2 in the SAED pattern insert are characterized by overlapping (002)_C_/(101)SiO_2_ and (100)_C_/(200)SiO_2_ diffraction, respectively [26].

A truly ubiquitous, ambient PM species was observed to be aggregates of carbon nanotubes and related fullerenic particles/polyhedra which occurred in many airborne (outdoor) samples as illustrated typically in Figs. 3 and 4. Note the characteristic dark-field image for the carbon nanotubes and fullerenic polyhedra, dominated by the graphite (002) and (100) reflections ((0002) and (10 1̄ 0) hexagonal Miller-Bravais notation [43]) noted in the SAED pattern insert in Fig. 3. More complex aggregates composed of mixtures of carbon nanotubes and related polyhedral forms, and silica (sand) nanocrystals, were also frequently observed, as illustrated typically in Fig. 5. These complex aggregates may form by eletrostatic attraction of airborne silica (sand) nanocrystals to the carbon nanoparticle aggregates shown in Figs. 3 and 4. Because of the small, mean aggregate sizes for this PM (Figs. 3 and 4), long airborne residence times of months or years are possible [47], allowing for complex aggregation shown in Fig. 5. It should be noted in Fig. 5 that the dark-field image in Fig. 5(b), slightly reduced in magnification and shifted from the bright-field TEM image in Fig 5(a), is dominated by silica (SiO_2_) nanocrystal diffraction. The prominent SiO_2_ (101) and (200) diffraction rings overlap the graphite (002) and (100) diffraction rings, respectively; represented by the corresponding arrows marked 1 and 2 in the SAED pattern superimposed on the bright and dark-field images in Fig. 5. It can be noted that the left portion of the dark-field image in Fig. 5(b) is particularly dense with SiO_2_ nanocrystals which range in size from about 1 nm to 5 nm, while the carbon nanotubes and other fullerenic polyhedra composing the aggregate, as shown in Fig, 5(a), range form 5 nm to 20 nm in diameter. These features are more readily observed in the carbon nanotube and related carbon nanocrystal aggregates shown in Figs. 3 and 4. Another feature of some note in Fig. 5 is the occurrence of many nanoparticle fragments at the edge of the aggregate in Fig. 5(a), indicative of the relatively weak binding of the primary particles in the aggregate.

**Figure 6:**
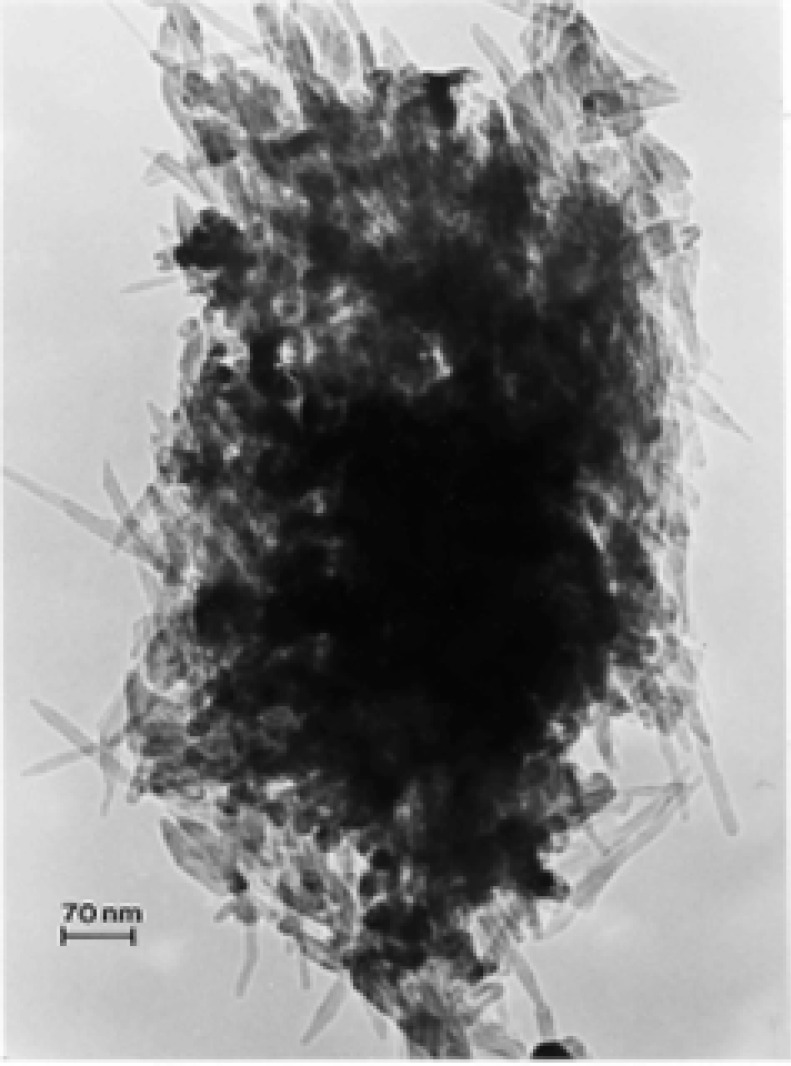
Typical PM example (d_p_ ≅ 1μm: 1.5μm × 0.6μm) of carbon nanotube / nanocrystal polyhedra aggregate collected from the roof-top exhaust of a natural gas water heater.

Atmospheric carbon nanoparticle aggregates have been demonstrated to be a common exhaust component in all natural gas and related methane-series fuel combustion [26, 27], including home hot water heater and furnace heater stacks vented to the outdoor environment through roof vents, a variety of industrial gas furnace operations venting to the outdoor environment, as well as indoor gas combustion sources such a kitchen stove burners, and other related, mobile (outdoor) sources. Figure 5 typically illustrates a carbon nanotube aggregate collected from a home roof-top, natural gas water heater exhaust. These numerous combustion sources account for the outdoor, airborne carbon nanotube aggregates illustrated typically in Figs. 3 and 6.

While carbon nanotube aggregates such as those illustrated in Figs. 3 and 6 in the outdoor ambient air are ubiquitous in the sense that they are observed on essentially all outdoor sampling grids in the TEM, their number concentrations only average ∼10^2^–10^3^/m^3^.

These concentrations can be compared with measured concentrations of ∼10^10^/m^3^ for cigarette smoke PM collected in the thermal precipitator [45], bulk measured abundance of PM under normal outdoor background conditions of ∼10^10^/m^3^ [42], or peak concentrations of ∼10^11^/m^3^ in episodic events; with corresponding PM mass concentrations of ∼50 μg/m^3^ [49]. This is in contrast to PM (smoke) concentrations of up to 400 μg/m^3^ during the London smog episodes in the winters of 1958–1972 [50], or typical, allowable 8-h concentrations for industrial dusts in occupational environments which can range for 2 to 10 mg/m^3^ [39]. Average DPM mass abundance ranging up to 1.4 mg/m^3^ has also been reported in studies of confined volumes in underground mines [51].

In contrast, ambient mass concentrations of DPM in the environment (or similar, aggregated carbon spherule matter) were estimated to be <1 μg/m^3^ in our studies; with DPM or DPM-like material representing more than -half of the carbon represented in Fig. 2 (∼20%), while carbon nanotube aggregates in the various for ms illustrated typically by Figs. 3 and 6 also account for ≤20% of the outdoor, airborne carbon (Fig. 2). Graphite PM from sources such as brake-lining debris, etc. have been shown to account for <2% of the carbon in Fig. 2 [13, 26]. This represents the average, daily carbonaceous PM in the ambient (outdoor) El Paso, TX air; with some variance between winter and summer months.

### TEM Characterization of Indoor (Kitchen) Carbon Nanotube Aggregates

**Figure 7:**
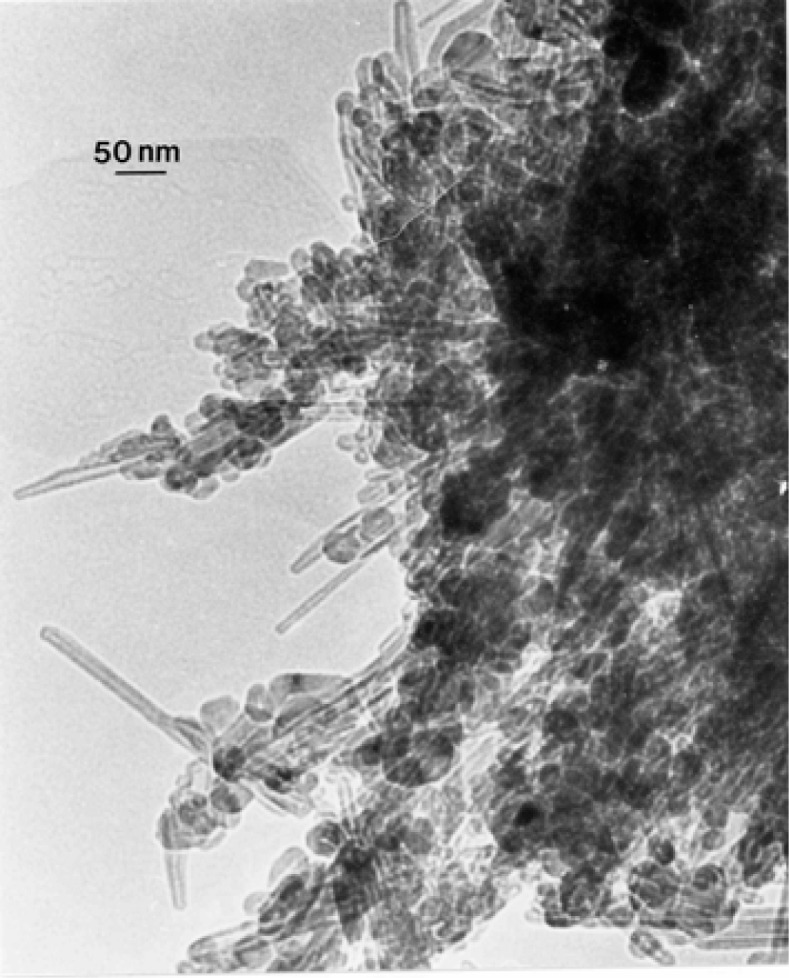
Large (∼2 μm) carbon nanotube aggregate collected from a kitchen natural gas burner exhaust stream. The estimated primary nanoparticle (MWCNTs and fullerene nanopolyhedra) number is ∼8000. The TEM image shows only about one-fourth of the total aggregate.

**Figure 8:**
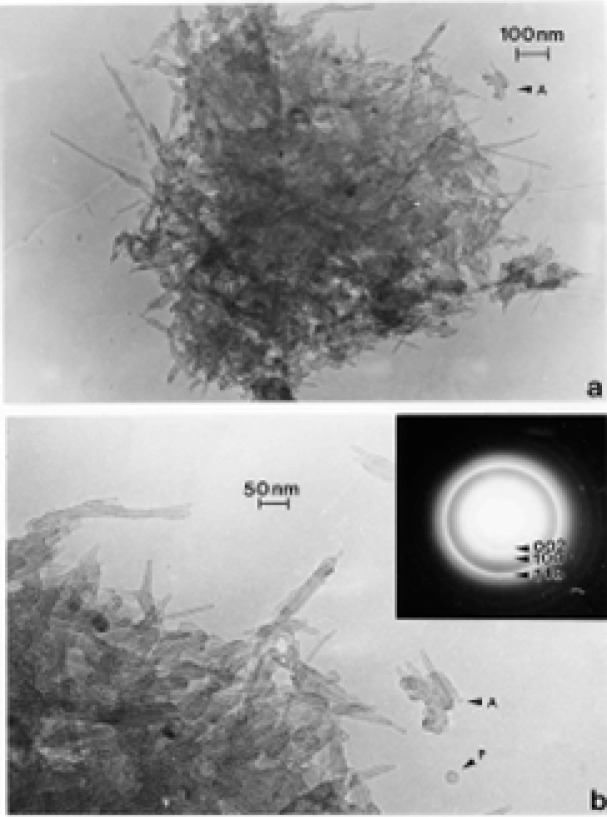
(a) Typical aggregate of carbon nanotubes and other fullerene polyhedra collected above a kitchen propane gas stove burner. (b) Magnified view of the aggregate in (a) showing a cluster of carbon (concentric) nanoshell structures (A) and a concentric, faceted, polyhedron (p). The SAED pattern insert show the principal graphite (graphene) reflections. The (002) diffraction ring corresponds to graphene (d) spacings of 0.34 nm. The propensity of carbon nanotubes of various length-to-diameter ratios (aspect ratios) relative to fullerene nanopolyhedra is roughly 9-to-1.

Figures 7 and 8 show typical examples of kitchen gas stove burners emission of carbon nanotube aggregates. Particle or aggregate concentrations in kitchens vary, but average ∼10^3^/m^3^ to 10^5^/m^3^ above gas burners; roughly 10^2^ to 10^3^ times the outdoor carbon nanotube aggregate number concentration noted above; but nonetheless a relatively low concentration. It can be noted in Figs. 7 and 8 that the carbon nanotube microstructures are essentially identical to those observed in various collection samples representing the outdoor air (Figs. 3 and 6). In addition, carbon or graphitic spherule aggregates resembling black carbon or DPM are also observed in kitchens with gas stoves.

### TEM Characterization of DPM, WPM, TPM,

**Figure 9:**
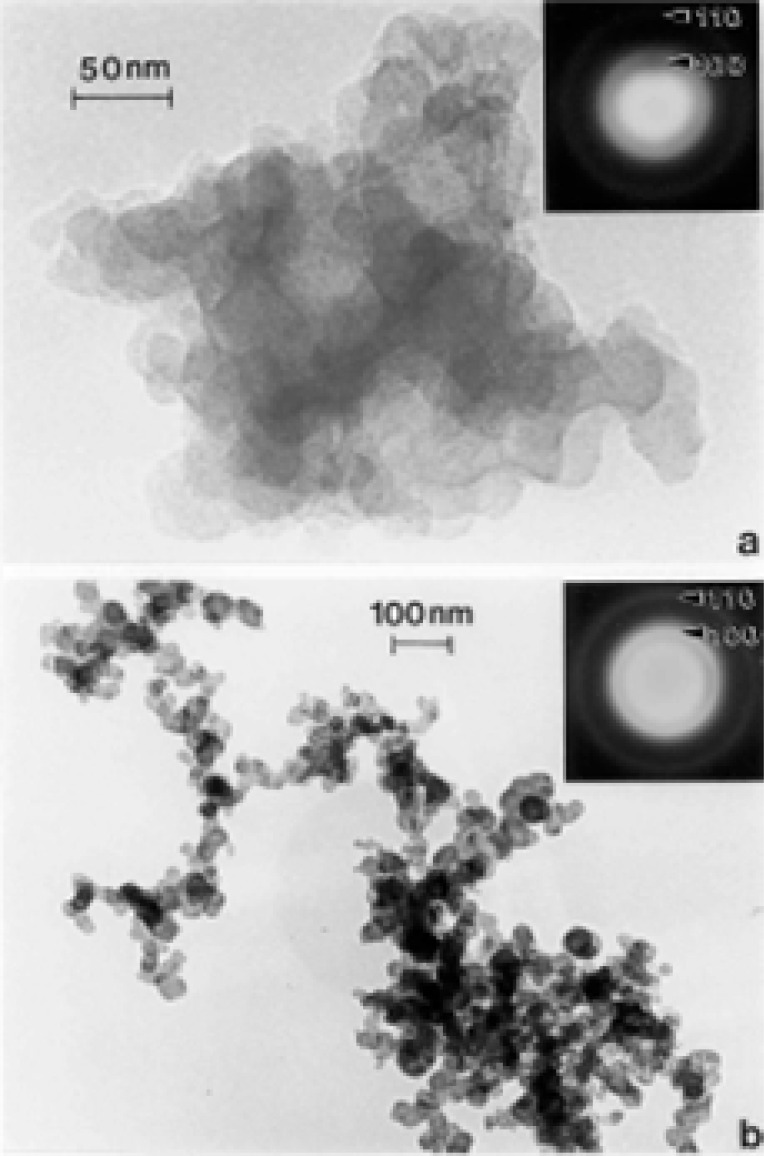
Examples of DPM collected in the vicinity of an area truck stop in El Paso, TX, USA. (a) Dense, BC-like aggregate of carbon spherules. Note diffuse diffraction rings in SAED pattern insert. (b) Complex, branched clusters of carbon spherules. SAED pattern insert shows crystalline diffraction rings [36].

**Figure 10:**
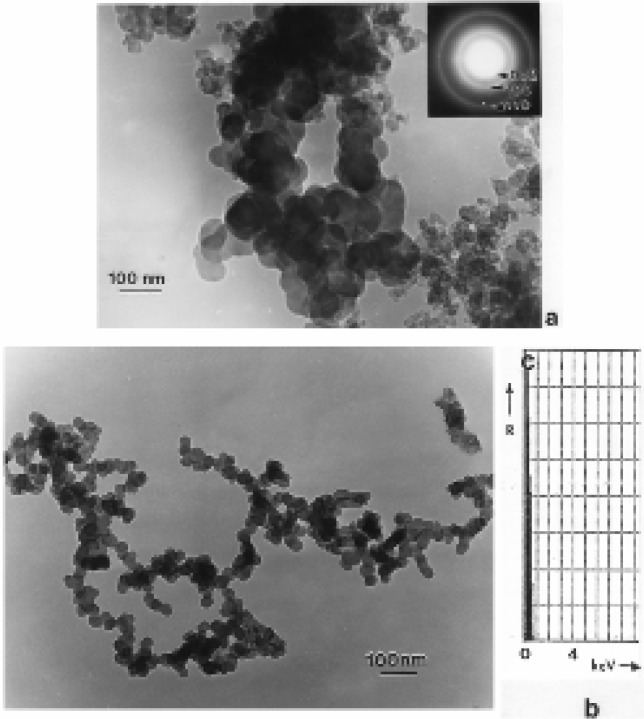
(a) DPM collected from a diesel bus location showing variations in carbon spherule aggregate structures (small and large spherule aggregates). SAED pattern insert shows well-defined graphite diffraction rings. (b) Complex, branched, carbon spherule aggregate characteristic of wood burning (WPM). Note similarity with DPM in Fig. 9(b). The EDS insert shows only carbon.

Figures 9 and 10(a) compare different DPM aggregates and similar types of branched, carbonaceous spherule clusters characteristic of wood burning or wood particulate matter (WPM) (Fig 10(b)) [52]. Of particular interest on comparing Figs. 9(a) and (b) and 10 is not only the variation in aggregate morphology, but also the variations in the degree of apparent crystallinity evidenced by comparing the SAED pattern inserts in Figs. 9(a) and (b) and Fig. 10(a); and especially the appearance of the principal graphite diffraction rings ((002) and (100)) indicated by the arrows marked 1 and 2, respectively.

Figure 9(a) represents a rather dense aggregate of carbon/carbonaceous spherules which are characteristic of non-optimized diesel soot or black carbon (BC) soot. The diffraction rings (SAED pattern insert) are very broad and diffuse (especially (100) and (110) in contrast to Fig. 9(b)); reminiscent of what is normally referred to as amorphous structure. Indeed, the spherule structure consists of very small graphite (or graphene) fragments which are packed together rather randomly (referred to as turbostratic structure). The individual spherules in Fig. 9(a) range in size from roughly 20 to 50 nm in diameter, while the mean aggregate diameter (d_p_) is roughly 300 nm. In contrast to Fig. 9(a), Fig. 9(b) shows a much less dense, branched-cluster aggregate with a mean diameter of ∼1.5 μm; with a maximum dimension of ∼2 μm corresponding to a radius of gyration (the density of primary particles or spherules about the centroid of the aggregate) of ∼1 μm. Although there is a difference in the magnification of the images in Fig. 9(a) and (b), there is clearly a difference in the spherule structure in Fig. 9(b) in contrast to Fig. 9(a) as evidenced by the more clarified or sharper (100) and (110) diffraction rings in the SAED pattern inserts as noted. This reflects the larger graphene fragments and thicker registry regimes (turbostratic structure) of these fragments in the spherules of Fig. 9(b) in contrast to Fig. 10(a) [53, 54]. (The spherule sizes in Fig. 9(b) range from 10 nm to 50 nm in diameter. Figures 9 and 10(a) represent essentially two types of DPM particle and aggregate microstructural features as well as graphitic, turbostratic structures which are related to the efficiency of the combustion process as well as other related phenomena. These were noted for WPM several decades ago [52].

Figure 10(a) shows a mixture of DPM aggregates with primary spherule particles ranging in size from ∼10 nm to 80 nm; with similar degrees of crystallinity in contrast to Fig. 9(b) as noted above. Figure 10(b) shows similar branched, carbon spherule aggregate morphology for wood combustion PM. Indeed the aggregate morphology in Fig. 10(b) is essentially indistinguishable from Fig. 9(b). The EDS spectrum inserted in Fig. 13(b) shows only carbon, and is characteristic not only of the EDS spectra for the other DPM aggregates shown in Figs. 9 and 10(a) but also the carbon nanotube aggregates shown in Figs. 3, 4 and 7 and 9; although some natural gas emission sources can contain very small amounts of sulfur.

Soot (as black carbon or DPM) generally forms under fuel-rich conditions in which hydrocarbon fragments or so-called polycyclic aromatic hydrocarbons (PAHs) which result by pyrolysis have a greater chance of colliding with other hydrocarbon fragments and growing, rather than being oxidized to CO, CO_2_ or H_2_O; especially when the C/O ratio exceeds unity. Soot can also form in flames of premixed hydrocarbons in air at C/O values ranging from 0.5 to 0.9 and in diffusion flames even in the presence of excess air (oxygen) [55]. Combustion temperature also plays a role and there is a complex competition in combustion processes between pyrolysis and oxidation. Consequently, a comparison of the variations in DPM (Figs. 9 and 10) in contrast to the variations in carbon nanotube aggregate PM (Figs. 3 and 8) can provide evidence that carbon (or carbonaceous aggregates) can range from classical soot, observable as black smoke, to carbon nanotube and related fullerenic nanoparticle aggregates or complex aggregates which are virtually in visible in clean-burning (blue flame) combustion [56]. It can be noted on comparing the various SAED pattern inserts in the images of carbon nanotube aggregates shown in Figs. 3 and 8 that these patterns are more crystalline (with sharper or better defined diffraction rings) than those for DPM (Figs. 9 and 10).

It is worth mentioning that whiles the measurement of mean aggregate diameters (d_p_) for the branched – cluster morphologies of DPM illustrated by Figs. 9(b) and Fig. 10(a) in particular is somewhat naive, it at least provides a geometric continuity in comparing the physical diameter s of airborne PM aggregates, particularly nanoparticulates. Large DPM aggregates such as those in Fig. 9(b) and Fig. 10(a) are often described as mass fractals and obey a power law relation between mass and the radius of gyration, r_G_ [22, 57, 58]. A so-called fractal or fractal-like dimension, D_f_, can be calculated when a log-log plot of the number of primary particles (or spherules), N, composing the aggregate versus 2 r_G_/d_s_ is performed: N = K (2 r_G_/d_s_)D_f_, where K is a dimensionless constant usually with a value of unity, and d_s_ is the primary particle or spherule diameter; which in Figs. 9 and 10 ranges from 10 nm to 80 nm. Note that 2 r_G_ is the maximum DPM aggregate dimension in Figs. 9(b) and 10(a), which of course is related to the mean aggregate (or particulate) diameter, d_p_ (Fig. 1). The fractal-like dimension, D_f_ has been observed to range from <2 to 3. Katrinak, et al. [22] found D_f_ to range from about 1.4 to 1.9 for DPM examined by TEM in the Phoenix, AZ (USA) air.

Clearly, DPM in its various aggregate morphologies and sizes is a ubiquitous anthropogenic PM in the outdoor air of essentially all cities, but carbon nanotubes and related fullerenic nanocrystal aggregates are even more ubiquitous as determined in this study, although the ambient carbon nanoparticle aggregate PM concentration is low. However, the concentration or particle abundance of this ubiquitous species can be two orders of magnitude greater in the indoor air, especially in kitchens where gas stoves are used for cooking as noted previously.

**Figure 11:**
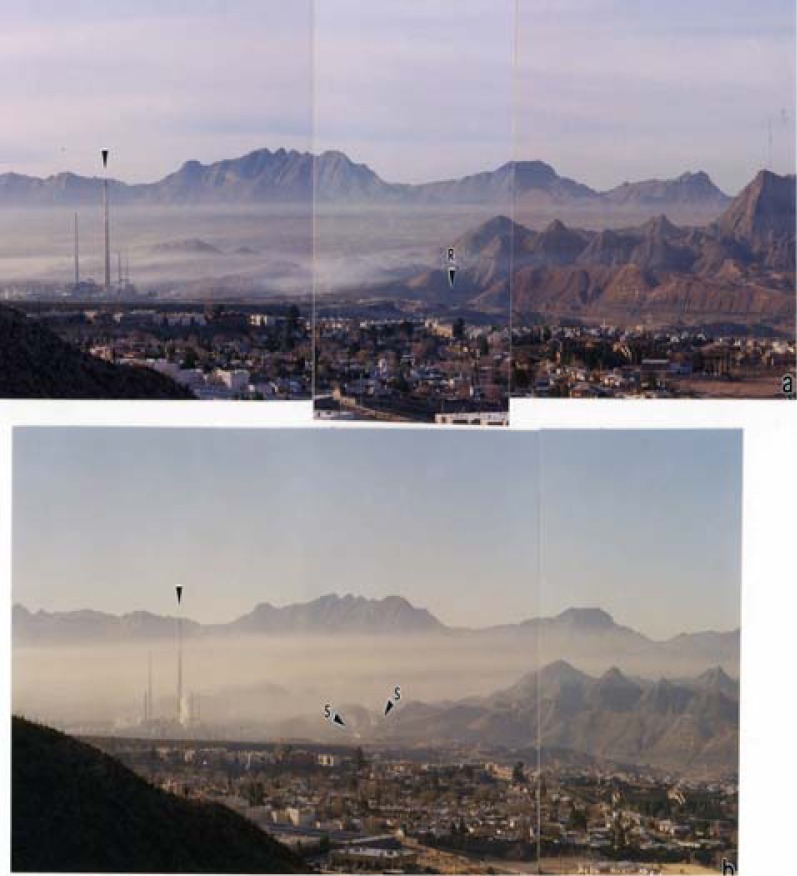
Early morning view (southwest) of particulate/smoke in version on the El Paso, TX USA/Juarez, Mexico border. (a) December, 2004. Arrow at left denotes reference at Asarco stack. R demotes the Rio Grande River. Juarez mountains are in the background. El Paso and Juarez city centers are at left out of view. (b) December, 1998. Arrows marked S show small smoke sources.

In addition to DPM and some varying preponderance of WPM in the El Paso ambient air, there is also often soot from burning tires which over the years have been a principle fuel in more than 300 brick-making operations in the Juarez, Mexico area. These operations also use various wood fuels, and such combustion regimes are especially prevalent at night and particularly noticeable in the late fall and winter months when thermal inversions are common. Figure 11 illustrates these atmospheric phenomena in a location just north of the El Paso city center, and the northern edge of Juarez. The Juarez mountains are in the background, and the Rio Grande river is noted (R) in Fig. 11(a). Some specific smoke (soot) sources are noted (S) in Fig. 11(b).

**Figure 12:**
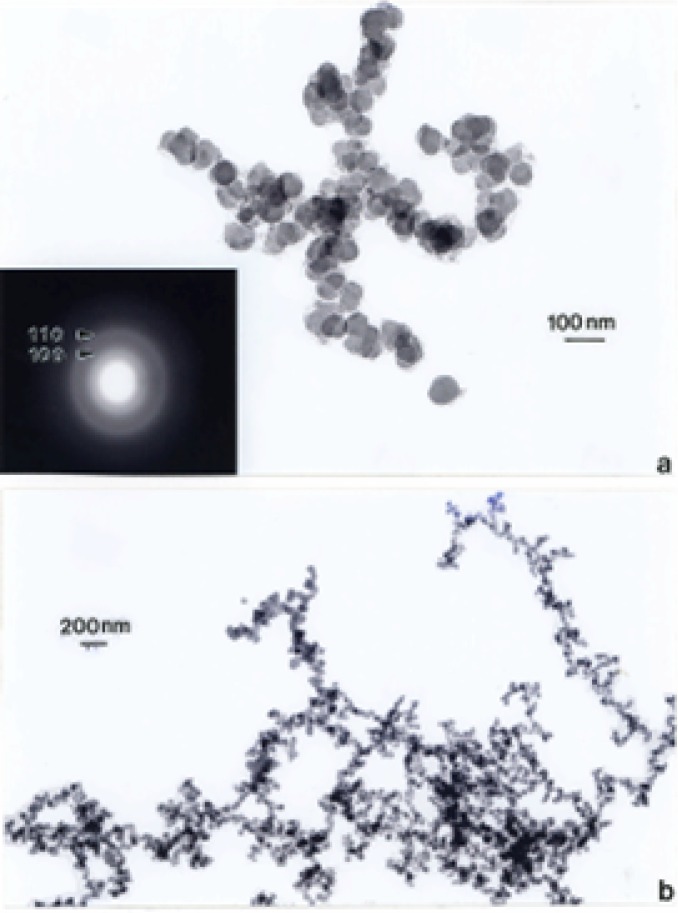
Particulate matter collected from a burning fire (TPM). (a) Small branched, aggregate fragment. The SAED pattern insert shows prominent graphite reflections. (b) Example of large, aggregated, branched particle. The image shows only about half of the aggregated particle.

While burning tire soot (or TPM) is not normally a significant or ubiquitous component of ambient air, little characterization of this soot has been reported. Figure 12 shows some examples of this soot collected from a burning tire by thermal precipitation. It can be noted on comparison with the DPM and WPM soot aggregates or branched spherule structures in Figs. 9(b) and 10 that TPM is micro structurally identical to DPM and WPM, and the SAED pattern insert in Fig. 12(a) is also very similar to those for DPM in Figs. 9(b) and 10(a). These carbonaceous PM are indistinguishable in a standard mass collection in the ambient air. On the other hand, burning tires emit a plethora of other toxic chemicals and usually never burn completely. Tires burn hotter than diesel fuel or wood, and is responsible for the large, well structured branched aggregates shown in Fig. 12. On average 1 tire pile fire is recorded daily in Juarez, Mexico which requires fire department response.

### Simple Microstructural Comparisons of Carbon Nanotubes and Soot PM

**Figure 13:**
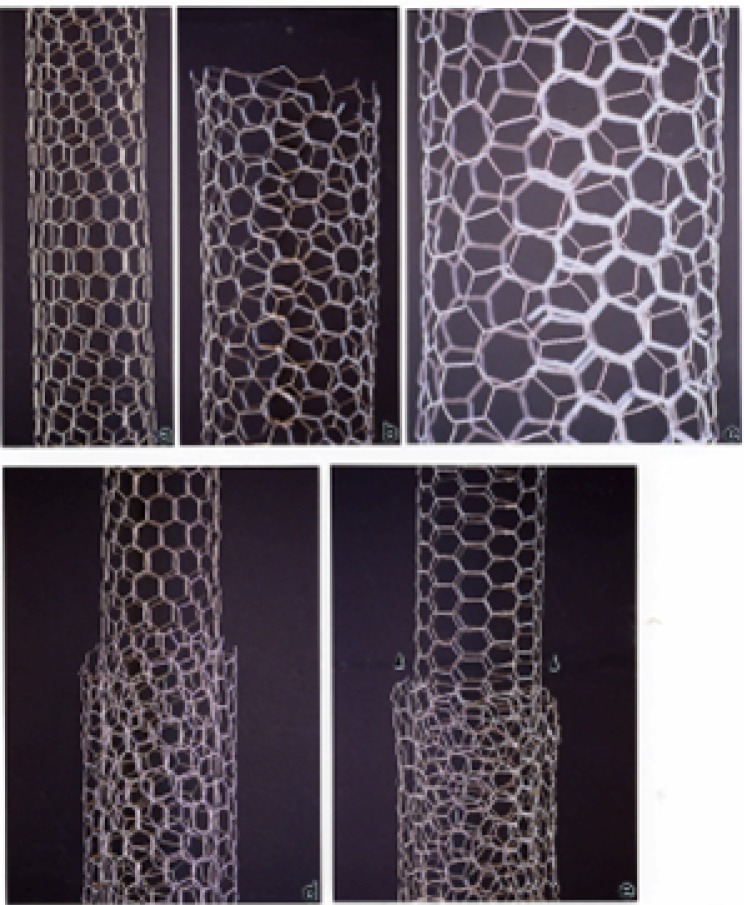
Simple chicken wire models of carbon nanotubes. (a) Zig-zag type single wall. (b) Chiral tube. (c) Magnified view of (b). (d) Chiral tube growing over the zig-zag tube in (a). (e) Chiral tube growing over an arm-chair type tube. Arrows indicate growth direction.

**Figure 14:**
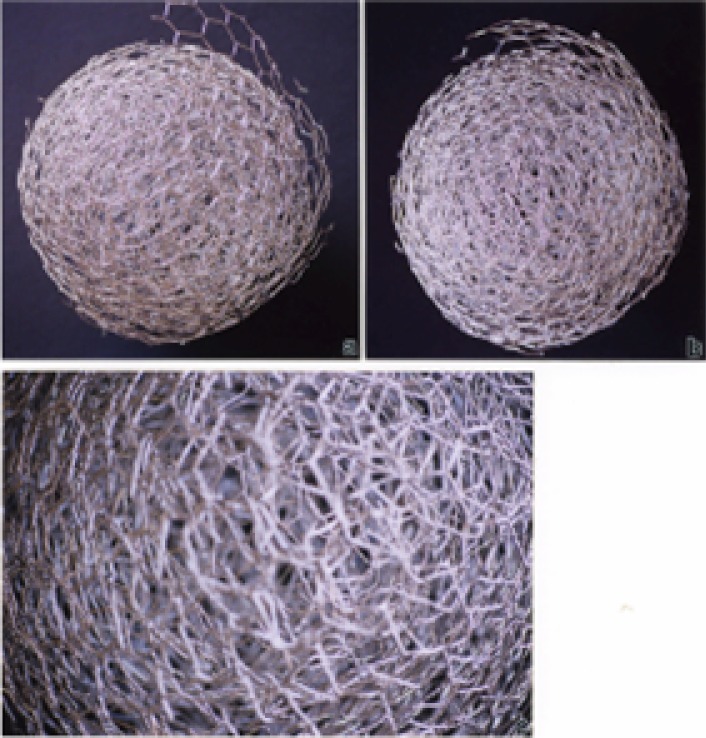
Chicken wire model of carbonaceous spherule formed by overlapping (turbostratic) graphene fragments. (a) and (b) show two views of the spherule. (c) is a magnified view of (b).

It should be apparent on comparing the TEM images and SAED pattern s for various car bon nanotube aggregates (Figs. 3 and 8) and the various soot aggregates and primary particles (Figs. 9, 10, and 12) that these PM are fundamentally and micro structurally distinct, despite the fact that they may be variously represented by irregular, variously extensive, and coincident or concentric graphene fragments or sheets. These fundamental micro structural features are illustrated in simple “chicken wire” constructs shown in Figs. 13 and 14, representing carbon nanotubes and turbostratic graphene spherules, respectively.

The tubes or tube segments shown in Fig. 13(a) to (c) represent single-wall zig-zag (Fig. 13(a)) and chiral types (Fig. 13(b) and (c)) which are the basis or substrates for multi-wall carbon nanotubes illustrated in Fig. 13(d) and (e) which show chiral tubes growing over zig-zag (Fig. 13(d)) and arm-chair (Fig. 13(e)) tube types [56, 59–61]. Figure 14(a) and (b) show corresponding views for a turbostratic-like soot spherule. These spherules aggregate into the fractile-like, branched clusters shown in Figs. 9, 10, and 12. The graphene fragments can also be considered in some instances to be polycyclic aromatic hydrocarbons of various molecular weights and these PAH species are often observed to be adsorbed as distinct species on the spherule surfaces. However, BC, with no adsorbed PAH components is identical in structure and characteristically represented by Fig. 14. It is therefore a common surrogate for in-vitro, cytotoxicity assays [39] and is characteristic of many indoor collections as well [56].

Of course the carbonaceous spherules represented in Fig. 14 can be composed of mixtures of fullerenes, including C_60_, intermixed with the turbostratic, graphene fragments, and variations in these types of spherule structure have in fact been observed by high-resolution TEM [53, 54]. Fullerenes and concentric fullerene polyhedra also form along with the multiwall carbon nanotubes and are aggregated with them in combustion sources as shown in the collected samples shown in Figs. 3 and 8 because both will nucleate from a fullerene-related template [61].

It is also important to recognize that, especially in the outdoor (ambient) air, there are not only complex mixing and aggregation of carbon nanotubes with nanosilica and other nanoparticulates as shown in Fig. 5, as well as adsorbed gas species and PAHs to other carbonaceous nanoparticle aggregates, but also pohotochemical, thermochemical, and related atmospheric transformations [62], and moisture (or water-related) degradation or alterations of carbon nanotube structures [28]. Sunlight, ozone, hydroxyl radicals, and other environmental or atmospheric components may complicate the biological effects observed in cytotoxic assays or other controlled in vivo experiments. Consequently, not only do a variety of synergistic atmospheric phenomena complicate the health effects of nanoparticulates, but microstructural and microchemical transformations may add to these complications.

### Cytotoxicity Assays

**Figure 15:**
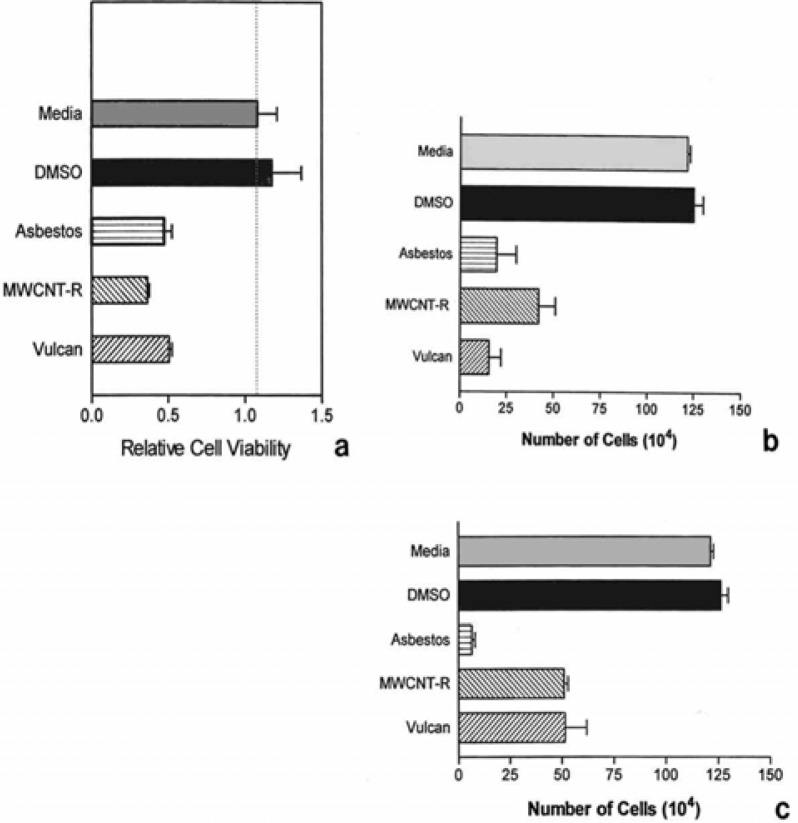
Comparative cytotoxicites of chrysotile asbestos surrogate BC, and surrogate carbon nanotube aggregates (MWCNT-R) to murine macrophage cells at a concentration of 5 μg/mL. (a) 2 days, (b) 7 days, (c) 14 days.

Figure 15 summarizes cytotoxicity assays for the surrogate BC and the MWCNT-R surrogate for anthropogenic carbon nanotube aggregates compared to the chrysotile asbestos nanotube material. Figure 15(a) shows the relative cell viability at 5 μg/mL after 2 days for the murine macrophage cells while Fig. 15(b) and (c) shows the actual cell counts after 7 days and 14 days respectively. While the BC and MWCNT-R surrogates are slightly less cytotoxic than the chrysotile asbestos, all of the experimental materials are observed to be cytotoxic. The cytotoxicity, or ability to kill macrophage cells increases with time for the chrysotile asbestos; while the surrogate nanomaterials exhibit a more constant killing ability. Light microscope examination of the cells killed in the chrysotile asbestos indicated an apoptotic mechanism consistent with species previous observations of apoptosis and apoptotic induction of reactive oxygen by asbestos [63–66]. In contrast, light microscopic observations of cells killed by exposure to the MWCNT-R surrogate illustrated features of both apoptosis and necrosis. The implications of these differences may be especially significant for long-term exposure to carbon nanotube aggregates in the indoor air, and will be described in detail elsewhere [67].

### Clinical and El Paso City-Wide Survey Data Analysis

**Figure 16:**
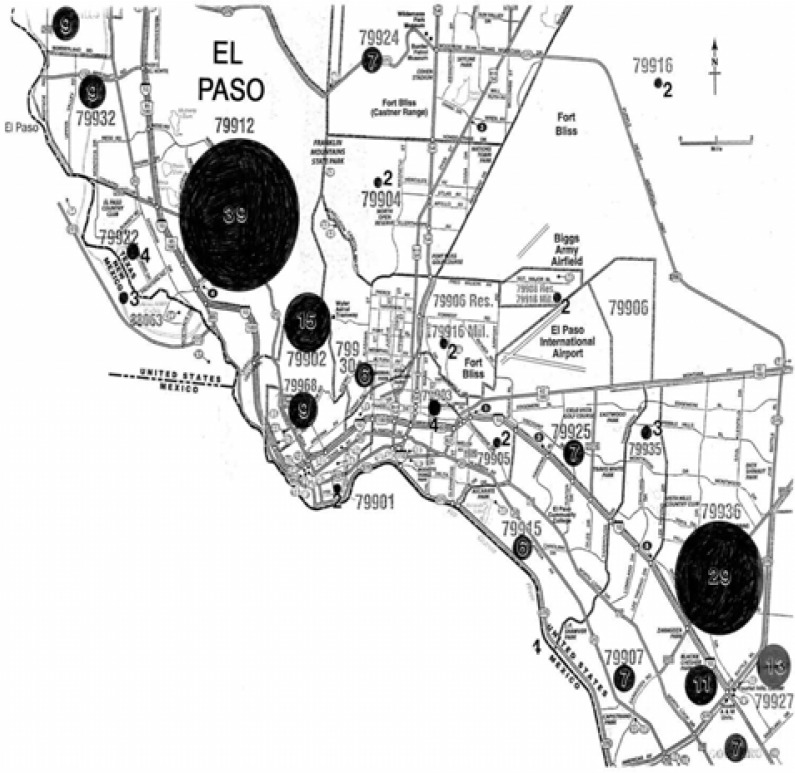
El Paso, TX, USA City map showing survey responses by zip code.

**Figure 17:**
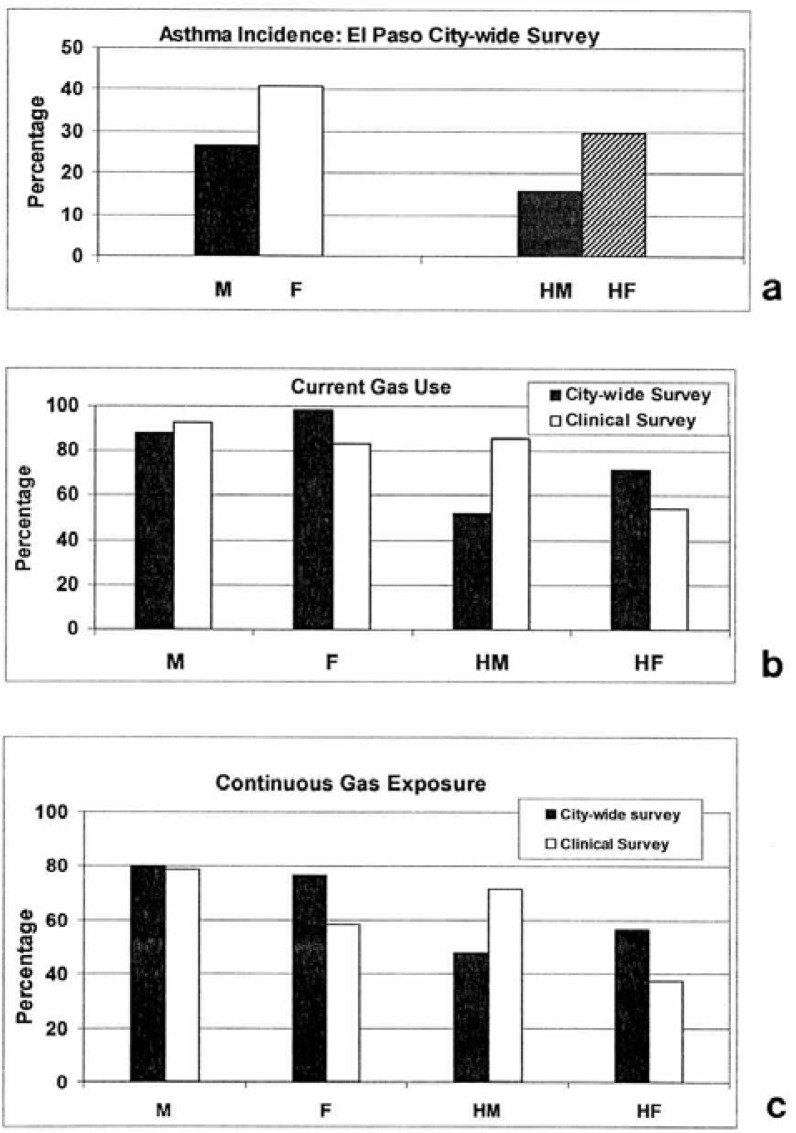
Clinical and El Paso city-wide survey data and comparisons. (a) City-wide asthma incidence (M-males, F-females; HM (Hispanic males), HF (Hispanic females). (b) Comparison of current (kitchen) gas stove exposure: clinical and city-wide respondents. (c) Comparison of continuous (or long-term) kitchen gas stove exposure: clinical and city-wide respondents.

Figure 16 illustrates the El Paso city-wide respondent locations by zip code. Of 200 respondents, half were male and half female. The distribution of the respondents is fairly uniform throughout El Paso. Figure 17(a) compares all male and female respondents with Hispanic male and female respondents who indicated some degree of asthma. It is interesting to note that the incidence of asthma is higher amongst female respondents, and the difference is greater between female and male amongst Hispanics where 30% of Hispanic females are asthmatic in contrast to 16% Hispanic males. The average asthma incidence for all male and female respondents in El Paso is roughly 34% (Fig. 17(a)) which is roughly 6 times the national average, but somewhat consistent with the fact that El Paso ranks 6^th^ for asthma incidence in the U.S. (Table 1). It was also found that of the 200 male and female respondents throughout El Paso (Fig. 16), 35% of males and 33% of females were smokers. More interesting was the fact that amongst the 34% asthmatics (both male and female) 51% were smokers. This compares with only 28% of smokers amongst the 38 clinical respondents, all of whom had some degree or category of asthma: 10% severe, 36% moderate, and 54% mild.

Figure 17(b) compares the El Paso City-wide and clinical respondents who have been continuously exposed to kitchen gas stoves (both natural gas and propane gas). The City-wide results for all male and female respondents were similar (80% male versus 77% female) while the clinical data showed a larger difference: 79% versus 58%, respectively. Correspondingly, current gas stove use in El Paso as shown in the data compared in Fig. 17(c) indicates that roughly 90% of all respondents currently use gas. The trends and differences between the City-wide respondents in contrast to the clinical respondents shown for continuous gas exposure in Fig. 17(b) are also shown for current gas exposure in Fig. 17(c). City-wide in El Paso, the use of gas is higher amongst Hispanic females than males. 72% of Hispanic females currently use or are exposed to gas kitchen stoves in contrast to 53% of Hispanic males. This is in contrast to 30% of Hispanic females City-wide with asthma as compared to 16% of males (Fig. 17(a)).

Table 1 compares asthma incidence, worst cities rank for asthmatics, Hispanic population percentage, homes with gas, city population (1990), estimated % gas exposure as a percent of the population, and other comparative, environmental-related parameters. It can be noted that while El Paso ranks 6^th^ for asthma incidence, it ranks 67 for worst asthma cities in the U.S. Correspondingly, Tucson, which ranks first for asthma incidence ranks only 61 for worst asthma cities. This occurs because of the migration of asthmatics over at least 3 decades to favorable city locations, and the fact that there is a hereditary (atopic) asthma factor. It is of interest to note that the estimated gas use or exposure in Table 1 (90%) is consistent with the average, current gas exposure shown in Fig. 17(c). However, Phoenix, which ranks third for asthma incidence and 14 for worst asthma cities in the U.S. has a gas use only about half that for El Paso and the other asthma high-incidence cities (Albuquerque and Tucson in Table 1). Table 1 also indicates the highest Hispanic population percentage in El Paso, and this is also consistent with the asthma incidence percentage shown in Fig. 17(a).

Figure 17 and Table 1 illustrate the complexity in studying asthma triggers or causes in cities like El Paso which are ranked as high asthma incidence areas. But the fact that there is both short term and longer-term cytotoxic response for carbon nanotube aggregates warrants a more systematic and concerted investigation especially of human long-term exposure to carbon nanotube aggregates in the environment. The important issue is the focus on a single nanoparticulate species which has been demonstrated to pose adverse health effects, especially respiratory health effects.

## Discussion

The uncertainty introduced by the interconverting PM mass concentration and particle number concentration combined with the fact that mass abundance in many cases is likely to be a surrogate for the real health effect agent complicates the etiology for many respiratory ailments, particularly asthma. This is especially true for studies of the effects of DPM in terms of the particulate (or aggregate) morphologies and sizes, as implicit on comparing Figs. 9, 10 and 12 in contrast to adsorbed PAHs or other DPM-PAH complexes characteristic of diesel exhausts (DE); as well as prospects for WPM-PAH or TPM-PAH complexes. Indeed, combustion particle characteristics investigated in toxicology studies vary widely [39], including DPM and DE, which has been shown to cause lung tumors in rats [21]. Zhiqiang, et al. [3] contend that DPM with the carbonaceous skeleton are carriers by which the particle-bound PAHs are transported deep into the human respiratory system. Recent work by Arrieta, et al. (68) involving rich PAH extracts (such as benzo [a] pyrene or BaP) from PM_10_ obtained from filters in the Paso del Norte air shed (which includes the El Paso, TX, USA, Juarez, Mexico, and Sunland Park, NM, USA region) indicated significant biologic activity, and a cancer risk of 5–12 cases per 10^5^ population.

Only a few PAHs are strongly carcinogenic and in the rankings of carcinogenic activity indices benzo [a] pyrene ranks 34 in the context of dibenzo [a,1] pyrene which ranks 123 and 7, 8, 12-trimethylbenz [a] anthracene which ranks highest at 147 [69]. Furthermore, the PAH content of WPM and especially TPM can contribute significantly to the El Paso/Paso del Norte air shed studies of Arrieta, et al. [68]. In addition other additive or synergistic interactions are also important in DPM effects, such as accompanying gas concentrations such as CO and especially ozone. For example, in a recent study by Kafoury and Kelley [70] involving IL-8 production by exposed epithelial cells (in vitro), DPM alone produced 21 pg/mL IL-8 in contrast to ∼117 pg/mL for DPM + ozone. Of course similar synergistic effects could occur for cigarette smoking in homes with gas appliances. Synergistic effects of cigarette smoking and long-term asbestos ingestion have also been documented [71].

There have not been any corresponding studies of PAHs associated with carbon nanotube or related fullerenic nanoparticle aggregates, either in outdoor or indoor environments. In contrast to the DPM, these complex carbon nanoparticle aggregates have considerably different microstructures (Figs. 13 and 14) which could create different PAH complexes if indeed PAH production is a significant cohort in the cleaner source processes associated with natural gas, propane, or related combustion, both stationary and mobile; indoor and outdoor.

While ultrafine particles account for less than 1% of general outdoor mass abundance of PM, they account for a significant fraction of the particle abundance or number concentration [38]. Furthermore essentially 80% of PM is aggregated and ∼95% of the primary particles composing these aggregates are <1μm in average diameter ; 57% of the primary particle components are <100 nm in diameter. While bulk, filter collected PM number concentrations for severe health considerations are often measured to be 10^11^/m^3^, these number concentrations are not characteristic of long-term exposures nor are they specific to PM chemistries, crystallinity, size or morphology. Furthermore, since the greater propensity of fine and ultrafine PM is aggregated and often susceptible to disaggregation during respiration [26], the epidemiology of respiratory health issues in particular is complicated, and PM toxicity or toxicological assessments, especially as they may apply to human respiratory health, are even more uncertain. In fact, toxicological studies tend to show that a short-term over abundance (mass abundance) of numerous PM agents instilled into mouse or rat lungs is not as compromising as smaller instillation doses administered over longer times. Indeed, asthma and other respiratory ailments are classically longer-term. In addition, these health effects are increasing at an alarming rate as efforts to decrease the overall atmospheric (outdoor) PM worldwide is also accelerating [1].

It is interesting to note that atmospheric BC measurements over the Southwestern U.S., and including the El Paso region, have produced mass concentrations of ∼0.15 μg/m^3^ in recent years [72, 73]. Correspondingly, the graphitic carbon content of snow measured in the same region of the U.S. was found to be between 2.2 and 25 μg/L of snow melt water in contrast to Greenland ice core samples where the graphitic carbon contents were found to be 2.5 and 1.1 μg/L for age-dated ice core melt water having approximate ages of 4000 and 6000 years, respectively, [74]; Murr, et al. [75] have also recently observed carbon nanotube aggregates essentially identical to those observed in contemporary outdoor air (e.g. Fig. 5) in a 10,000 year-old Greenland ice core, where the corresponding carbon nanotube aggregate content of the meltwater was estimated to be about 0.1 μg/L. These results suggest that while the anthropogenic carbonaceous contribution in the atmosphere may not be generally (globally or regionally) significant, the occurrence of carbonaceous nanoparticulates such as carbon nanotube aggregates in the indoor environment may be more significant, and the associated long-term exposure phenomena may be a larger contributor to respiratory health effects, particularly asthma.

Asthma is a chronic lung condition which causes difficulty in breathing due to airway narrowing or obstruction when they are irritated. Airway narrowing or obstruction is caused by inflammation or broncho constriction due to encircling airway muscle tightening or spasms. Allergens and other environmental triggers can also emulate these conditions through mediator production. The tendency to be allergic is inherited while a specific allergy response is not. The allergic reaction in the immune system can be by ingested, inhaled, or contacted allergens. The underlying cause is unknown while 3 to 5% of adults and 7 to 10% of children are affected. Since 1979 the rate of asthma has increased 60%. Ambient air particulates are potential triggers for asthma although it is unknown what specific environmental phenomena contribute to asthma in those with a genetic (or inherited) predisposition.

Many factors may influence the pathogenicity of airborne particulate matter, especially nanoparticulates: morphology, surface area and chemistry (including adsorbed species), and crystallinity and crystal structure. Little is known about the interaction and exposure with bronchoalveolar lining fluid constituents, especially in asthmatic humans [76]. While diverse hydrocarbon-based air pollutants may exacerbate existing allergic disease, there is no strong evidence that they cause asthma [77]. One of the more recent studies involving the exposure of healthy and mildly asthmatic human volunteers to nanoparticulate (∼25 nm) BC (at concentrations of 10 μg/m^3^ or 25 μg/m^3^) showed no detectable changes in airway, systemic, or cardiovascular electrophysiologic endpoints [78].

In addition to prospects for particulate matter influencing asthma symptoms, dysregulated immunity has also been cited as a factor because of the fact that epidemic asthma has occurred during decades of improved hygiene and reduced childhood infections [79, 80].

As noted earlier, the causal association between particulate matters, especially nanoparticulate matter, is related to mechanisms of PM toxicity and cytotoxicity which seem to ultimately involve some form of mediated generation of reactive oxygen species (ROS) [2] which underlie the inflammatory effects of PM. Nanoparticulates which penetrate the interstitium contact the interstitial macrophages and release ROS. These can enter the blood stream and lead to cardiovascular dysfunction [2]. Oxidative stress or ROS production has also been discussed as the basis for asbestos carcinogenicity [71, 81] as well as carcinogenesis of polycyclic aromatic hydrocarbons (PAHs) [82]. Even nanoparticulate C_60_ has been described as a potent generator of singlet oxygen which is known to be a highly cytotoxic species [83].

It is of interest to note that Polansky [84] has described exogenous events that move a biological system from “good health” to “chronic disease” as disruptions which are unknown for most chronic diseases, including asthma. However, Polansky [84] has correspondingly developed a theory that micro-competition with foreign DNA, which can be considered to be characterized by excessive oxidative stress, causes all chronic disease; including asthma.

While reactive oxygen species can provide a fundamental cause for diseases like asthma, the mechanism for producing inflammatory mediators in respiratory tract cells appears to be multifaceted. That is, there are numerous pathways or agents for producing ROS, and combinations or synergistic effects may complicate the onset of asthma in particular. For example, Fig. 15 illustrates that BC and multi-wall carbon nanotube aggregates, as surrogates for soot-related or combustion-related nanoaggregates and nanoparticulate PM, are cytotoxic and could produce inflammatory mediators. Soot with adsorbed PAHs and PAHs alone are able to produce ROS [68]. Transition metals (Ni, Co, Ti, Fe, Cu especially) have been associated with carcinogenicity and hypoxia [85] and transition metal components of fine and ultrafine particulate matter have been hypothesized to be important factors in toxicity and potential, adverse health effects [86, 87]. Vallyathan, et al. [88] have, among others, shown that during phagocytosis of inhaled particles H_2_O_2_ is produced which in the presence of iron and copper ions is converted to the potent, oxidizing hydroxyl radical through the Fenton reaction. Correspondingly, ultrafine PM inhibit phagocytosis more than mass equivalent fine PM, and there is good toxicological evidence that nanoparticulate matter causes lung inflammation even for relatively low toxicity materials [89].

Just as many environmental agents (pollen, bioaerosols, chemicals, PM, including mineral dusts [90], etc. trigger asthma attacks and other respiratory trauma [91] the cause of asthma also seems to be similarly complex or multifunctional. In addition, strong hereditary factors also complicate the specific causes or contributing PM and synergistic issues. As illustrated in Table 1, high incidence cities in the southwestern U.S. may have as much to do with migration and heredity as environmental PM or related factor s. Clearly, combustion products, including fly ash, soot of various kinds (Figs. 9, 10, 12), carbon nanotube aggregates both indoor and outdoor (Figs. 3 and 7), and their accompanying gaseous cohorts (CO, NO_x_, etc.) [92]; all contribute to adverse health effects and should be avoided, especially in the context of continuous or long-term exposure, even at very low concentrations characteristic of carbon nanotube aggregates above gas burners in kitchens.

## Conclusions

- 93% of the PM, fraction in the El Paso, TX air is crystalline while 80% of this PM is aggregated. 57% of the aggregated PM is composed of primary particulates with mean diameters less than 100 nm.
- 42% of the El Paso, TX PM_2.5_ contains carbon and of this PM roughly half represents anthropogenic soot: diesel, wood and tire combustion products and brake lining debris. The other half is essentially carbon nanotube aggregates (∼10%) which are ubiquitous in the ambient (outdoor) air even at nominal concentrations of 10^2^ to 10^3^/m^3^, and carbonates (∼5%). These nanoparticle aggregates originate from natural gas and other gas combustion sources venting to the atmosphere: water heaters, furnaces (both residential and industrial), power generation plants, etc. Aggregates of carbon nanotubes, fullerene polyhedra, and nanoparticulate silica (SO_2_) are a common occurrence in the ambient air.
- The indoor (kitchen) number concentration of carbon nanotube aggregates is approximately 10^3^/m^3^ to 10^5^/m^3^ during gas use (above the gas burners). Carbon spherule aggregates or soot, appearing like DPM or other complex, branched structures also occur at similar number concentrations.
- The fractile-like, branched, aggregated spherules of turbostratic or more regular (even) fullerenic graphene fragments are indistinguishable structures characteristic of diesel, wood, or tire combustion soot.
- In contrast to the irregular or turbostratic graphene fragment structure characteristic of anthropogenic soots, with primary spherule particle sizes ranging from 20 to 80 nm, carbon nanotube aggregates are variously mixed multiwall carbon nanotubes (ranging in diameters from 10 to 40 nm) and concentric fullerene polyhedra having similar diameters.
- Black carbon (BC) and a multi-wall carbon nanotube aggregate surrogate were demonstrated to be cytotoxic for a murine macrophage cell line for exposures ranging from 2 days to 2 weeks. Both a chrysotile asbestos control and the MWCNT surrogate effectively killed the macrophage cells but the MWCNT surrogate demonstrated both apoptosis and necrosis in contrast to the asbestos-induced apoptosis.
- Analysis of El Paso City-wide survey data indicated that 34% of all respondents were asthmatic; nearly 6 times the U.S. national average of 6%. Nearly an equal number of respondents were smokers but amongst asthmatics 51% were smokers. Correspondingly, only 28% of clinical asthma respondents were smokers. Approximately 78% of City-wide respondents were continuously exposed to gas stove use; slightly less for clinical respondents. The current gas use is considerably higher amongst Hispanic females in contrast to Hispanic males for both the City-wide survey data and the clinical survey data. Considering asthma migration and hereditary factors, gas use or exposure and asthma may be circumstantial rather than problematic.
- Because of the ability of various particulates, especially nanoparticulates, to induce reactive oxygen species as well as transition metal ions, adsorbed PAHs, and other gas-phase species, including CO, NO_x_, ozone, etc., respiratory adversities are characterized by intermittent and long-term exposures to a complex synergy of inflammatory agents, especially in a complex region like the Paso del Norte air shed.

## Figures and Tables

**Table 1:** U.S. City comparison

City**	*Asthma Prevalence U.S. Rank (2002)*	Worst Asthma^*†^ Cities Rank (2005)	*% Hispanic (1990)*	*Homes with Gas (1990)*	*City Population (1990)*	% Estimated Gas Exposure* Population: 1990 (%)	*Clear Days Ave. (1997)*	*Fugitive Air^†^ TCR 1996 (lbs.)*	*SO_2_ (1996) (lbs)*	*NOX (96) (lbs)*	*VOC^††^ (96) (lbs)*	*CO (96) (lbs)*	*PM_10_ (96) (lbs)*
ELP	6	67	74	136138	602951	90	192	134328	9026	32670	33929	142337	15509
ABQ	7	77	38	133838	426736	100	168	4848	4365	37505	36799	223214	86547
PHX	3	14	20	150326	1139793	53	210	641475	5971	154710	139195	536117	68244
TUC	1	61	28	105824	472385	90	194	105970	9805	49424	43436	218623	35286
LA	--	42	38	957619	3498138	100	143	4268754	27047	328380	415073	1726306	105181
SDG	--	82	25	110982	1168364	38	146	402010	5391	92144	113722	497027	88532
HON	--	--	7	7381	878044	3	90	318432	21380	32069	23994	166518	24239
OMA	24	45	4	117630	350602	100	111	615710	23477	42233	36334	166820	30239
BOS	--	66	5	93179	552519	67	98	23556	8495	28363	18606	140723	15957

* Estimated 4 persons per household: 4 × Homes with gas; %Gas Exposure = (4 × Homes with gas/population) (X100%).

† TCR (Toxic Chemical Release (lbs)) for fugitive air. All quantities are lbs/day (average) for 1996.

†† VOC: Volatile organic carbon

** Based on data in Molecular Similarity/Toxicity Model of Benzene Derivatives and a Similarity Analysis of Environmentally Impacted Regions, vol. II, Dissertation by Gabrielle Rum, University of Texas at El Paso, El Paso, TX, 1999.

In similarity studies involving these cities with 204 descriptors, El Paso was the most dissimilar of all while Phoenix was the most similar to El Paso.

This table represents only 11 of these 204 descriptors. ELP    El Paso ABQ  Albuquerque PHX  Phoenix TUC  Tucson LA  Los Angeles SDG  San Diego HON  Honolulu OMA  Omaha BOS  Boston

*† Asthma and Allergy Foundation of America asthma risk, medical, and prevalence factors ranking the worst cities for asthma sufferers. (web MD Medical News)
